# Heterophyllin B Ameliorates Lipopolysaccharide-Induced Inflammation and Oxidative Stress in RAW 264.7 Macrophages by Suppressing the PI3K/Akt Pathways

**DOI:** 10.3390/molecules23040717

**Published:** 2018-03-21

**Authors:** Chunjing Yang, Longtai You, Xingbin Yin, Yi Liu, Xin Leng, Wenping Wang, Na Sai, Jian Ni

**Affiliations:** School of Chinese Materia Medica, Beijing University of Chinese Medicine, Beijing 100029, China; 20160941191@bucm.edu.cn (C.Y.); ylt_svp@163.com (L.Y.); yxbtcm@163.com (X.Y.); 18795804002@163.com (Y.L.); 20160931927@bucm.edu.cn (X.L.); wangwenp6@163.com (W.W.); yxsaina@126.com (N.S.)

**Keywords:** Heterophyllin B, anti-inflammatory effect, anti-oxidative effect, anti-apoptosis

## Abstract

Heterophyllin B (HB), an active cyclic peptide, is a compound existing in the ethyl acetate extract of *Pseudostellaria heterophylla* (Miq.) Pax and exhibited the activity of inhibiting the production of NO and cytokines, such as IL-1β and IL-6, in LPS-stimulated RAW 264.7 macrophages. In addition, HB suppressed the production of ROS and the apoptosis induced by LPS in RAW 264.7 macrophages. The underlying mechanism was investigated in the LPS-induced RAW 264.7 cells. The results showed that HB decreased the level of IL-1β and IL-6 expression by qRT-PCR analysis. HB up-regulated the relative ratio of p-AKT/AKT and p-PI3K/PI3K as indicated by western blot analysis. In summary, HB inhibited the LPS-induced inflammation and apoptosis through the PI3K/Akt signaling pathways and represented a potential therapeutic target for treatment of inflammatory diseases.

## 1. Introduction

Inflammation is a biological reaction that occurs in response to tissue damage with the aim to remove harmful stimuli, including damaged cells, pathogens, or endotoxins like lipopolysaccharide (LPS) [1]. Inflammatory mediators such as reactive oxygen species (ROS), nitric oxide (NO), and interleukins (IL-1, IL-6) are overproduced when the macrophages are triggered. However, in high concentrations, inflammatory mediators have been implicated in the pathological processes involved in many inflammatory diseases, including rheumatoid arthritis, cardiovascular diseases, chronic hepatitis, pulmonary fibrosis, and inflammatory brain disease [2]. Thus, it is necessary to inhibit the excessive inflammatory response to reduce the damage caused by the inflammatory mediators [3]. 

**Figure 1:**
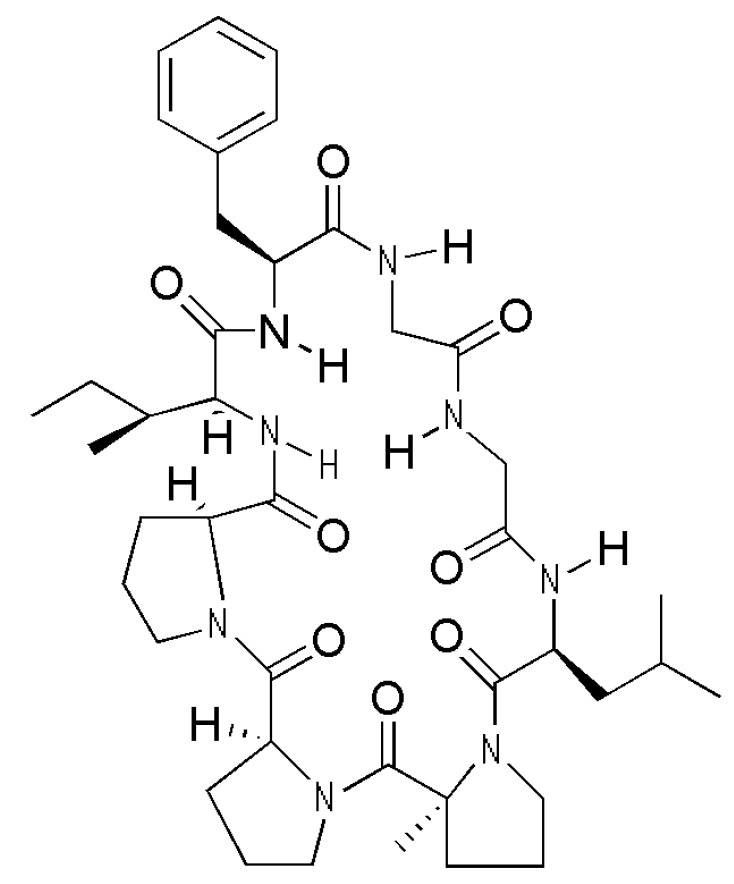
Chemical structure of Heterophyllin B (HB).

The ethyl acetate extract of *Pseudostellaria heterophylla* (Miq.) Pax exhibits anti-inflammatory activity. Heterophyllin B (HB, Figure 1) is a compound existing in the ethyl acetate extract of *Pseudostellaria heterophylla* (Miq.) Pax, which is often used in promoting the production of fluid in Chinese medicine [4]. HB is one of the active cyclic lipopeptides observed to exhibit antitumor, anti-inflammatory, antifungal, and antibacterial activities [5,6,7]. However, the effect of HB in association with LPS-induced inflammation and oxidative stress in RAW 264.7 macrophages has not been explored. Therefore, in this study, we attempted to determine the underlying mechanisms of its anti-inflammatory and anti-oxidative activities. 

## 2. Results

### 2.1. Effect of HB on Cell Viability

**Figure 2:**
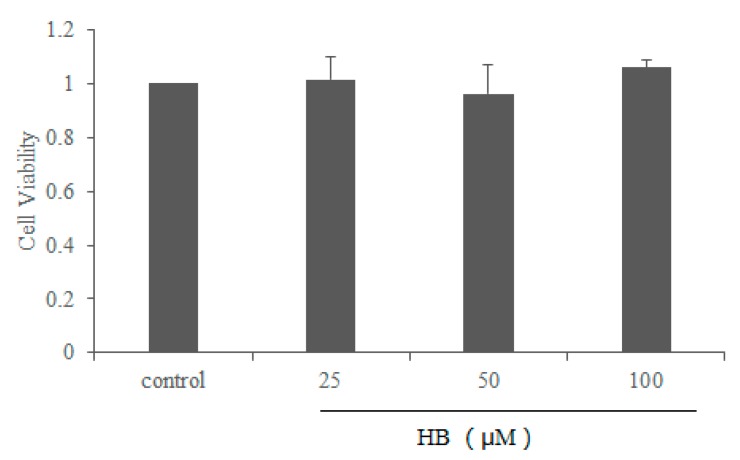
Effects of HB on the cell viability in RAW 264.7 Cells. RAW 264.7 Cells were cultured with different concentrations of HB (0, 25, 50, and 100 μM) for 24 h. The cell viability was detected by MTT assay. The values are presented as means ± SD of three independent experiments.

Compared with the vehicle controls, the results of the MTT assay demonstrated that HB at the concentration ranging from 0–100 μM had no cell toxicity (Figure 2). Thus, HB at concentrations 25, 50, and 100 μM was used in the subsequent experiments.

### 2.2. Effects of HB on LPS-Induced Generation of NO in RAW264.7 Cells

**Figure 3:**
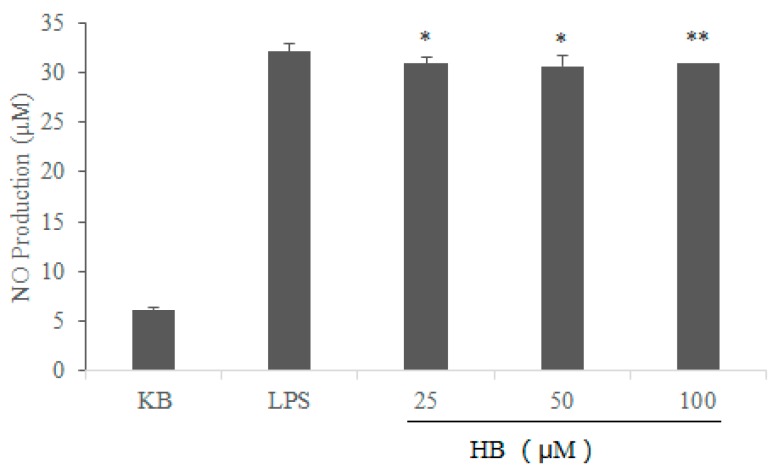
Effects of HB on LPS-induced NO production in RAW 264.7 Cells. RAW 264.7 Cells were pretreated with different concentrations of HB (25, 50, and 100 μM) for 1 h before treatment with 1 μg/mL LPS. After incubation for 18 h, the NO production analysis was detected by Griess test. The values are presented as means ± SD of three independent experiments. * *p* < 0.05, ** *p* < 0.01, compared to the LPS-treated group.

In order to investigate the effects of HB on LPS-induced inflammatory responses, we first examined the ability of HB to regulate NO production in response to LPS stimulation. The results showed that the level of NO increased significantly in LPS-stimulated cells when compared with the control group. However, HB reduced the production of NO in LPS-stimulated RAW 264.7 cells in a dose-dependent manner compared to the LPS group (Figure 3). 

### 2.3. Effects of HB on LPS-Induced Generation of IL-6, IL-1β in RAW264.7 Cells

**Figure 4:**
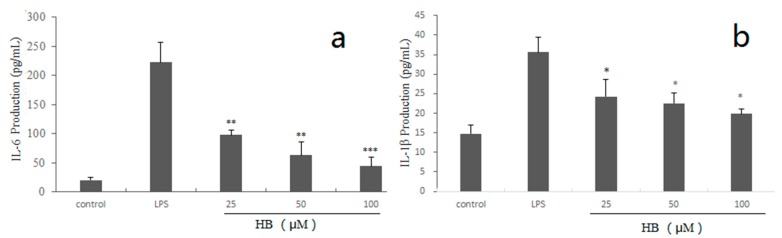
Effects of HB on LPS-induced production of IL-6 (**a**) and IL-1β (**b**) in RAW 264.7 Cells. RAW 264.7 Cells were pretreated with different concentrations of HB (25, 50, and 100 μM) for 1 h before treatment with 1 μg/mL LPS. After incubation for 18 h, the IL-6 and IL-1β production analysis was detected by ELISA kits. The values are presented as means ± SD of three independent experiments. * *p* < 0.05, ** *p* < 0.01, *** *p* < 0.001, compared to the LPS-treated group.

In order to examine whether HB exhibits anti-inflammatory activity by eliminating the cytokines (IL-6, IL-1β). Compared with the control group, the production of cytokines was significantly reduced after exposure to different concentrations of HB (Figure 4).

### 2.4. Effects of HB on LPS-Induced Generation of ROS

**Figure 5:**
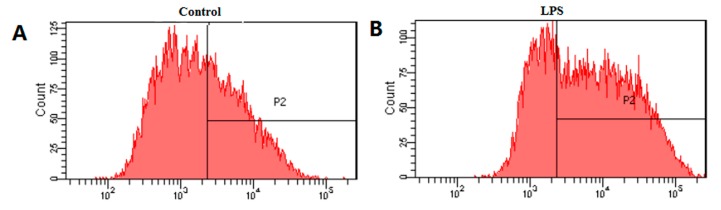

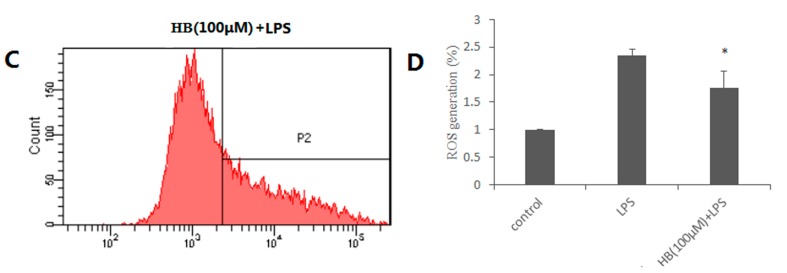
Effects of HB on LPS-induced ROS production in RAW 264.7 Cells measured by flow cytometry. (**A**) Control (**B**) LPS (1 μg/mL) (**C**) RAW 264.7 Cells were pretreated with 100 μM HB for 1 h before treatment with 1 μg/mL LPS for 18 h. (**D**) HB reduced the LPS-induced ROS production in RAW 264.7 Cells. Cells incubated with 1μg/mL LPS for 19 h were used as a positive controls. The values are presented as means ± SD of three independent experiments * *p* < 0.05, compared to the LPS-treated group.

Previous studies have shown that ROS generated mainly by the mitochondria can induce oxidative stress to promote apoptosis, leading to cell death [8,9]. In order to investigate whether HB induces oxidative stress, the effects of HB on intracellular ROS was measured. As shown in Figure 5, ROS generation was significantly increased by treatment with LPS and the increase was effectively attenuated in a concentration-dependent manner by treatment with HB.

### 2.5. Effects of HB on LPS-Induced Apoptosis in RAW 264.7 Cells

**Figure 6:**
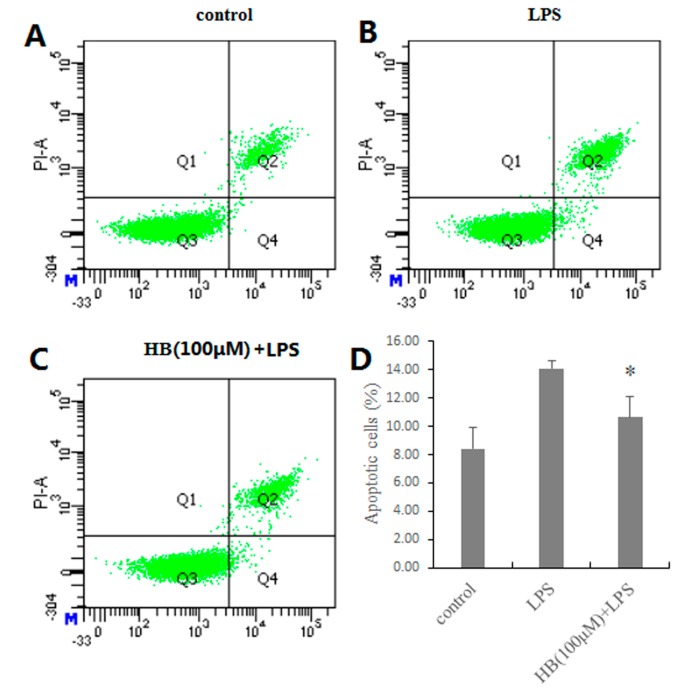
Effects of HB on LPS-induced apoptosis in RAW 264.7 Cells measured by flow cytometry. (**A**) Control (**B**) LPS (1 μg/mL) (**C**) RAW 264.7 Cells were pretreated with 100 μM HB for 1 h before treatment with 1 μg/mL LPS for 18 h. (**D**) HB reduced the LPS-induced apoptosis in RAW 264.7 Cells. Cells incubated with 1 μg/mL LPS for 19 h were used as a positive controls. The values are presented as means ± SD of three independent experiments * *p* < 0.05, compared to the LPS-treated group.

In order to determine whether HB inhibited the apoptosis of RAW 264.7 cells, we performed DAPI staining and flow cytometry assays. As shown in Figure 6, LPS-induced apoptotic nuclear fragmentation and condensation of chromatin were clearly observed by DAPI staining. Annexin V/PI double staining was used to quantify the apoptotic cells. Compared with the control group, the proportion of viable cells was significantly lower after exposure to LPS (1 μg/mL). Meanwhile, the proportion of early and late apoptotic cells significantly increased. Overall, these results clearly suggested that HB could inhibit the apoptosis of RAW 264.7 cells.

### 2.6. Effects of HB Treatment on LPS-Induced IL-6 and IL-1β Expression in RAW 264.7 Cells

**Figure 7:**
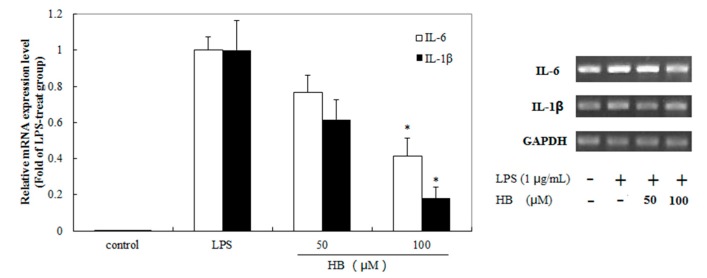
Effect of HB on LPS-induced expression of IL-6 and IL-1β in RAW 264.7 cells. RAW 264.7 cells were pretreated with 50 and 100 μM HB for 1 h before treatment with 1 μg/mL LPS. After incubation for 18 h, the expression of levels of IL-6 and IL-1β mRNA were measured by RT-PCR analysis. The data is expressed as mean folds of the mRNA expression versus LPS-stimulated group. The values are presented as means ± SD of three independent experiments. * *p* < 0.05, compared to the LPS-treated group.

In order to examine the anti-inflammatory effects of HB, we determined the levels of IL-6 and IL-1β expression after exposure to LPS-induced RAW 264.7 cells with HB (50 or 100 μM). The results showed that the expression of IL-6 and IL-1β mRNA was increased when RAW 264.7 cells were stimulated with LPS, which were suppressed by HB treatment (Figure 7).

### 2.7. HB Suppressed LPS-Induced PI3K and AKT Phosphorylation

**Figure 8:**
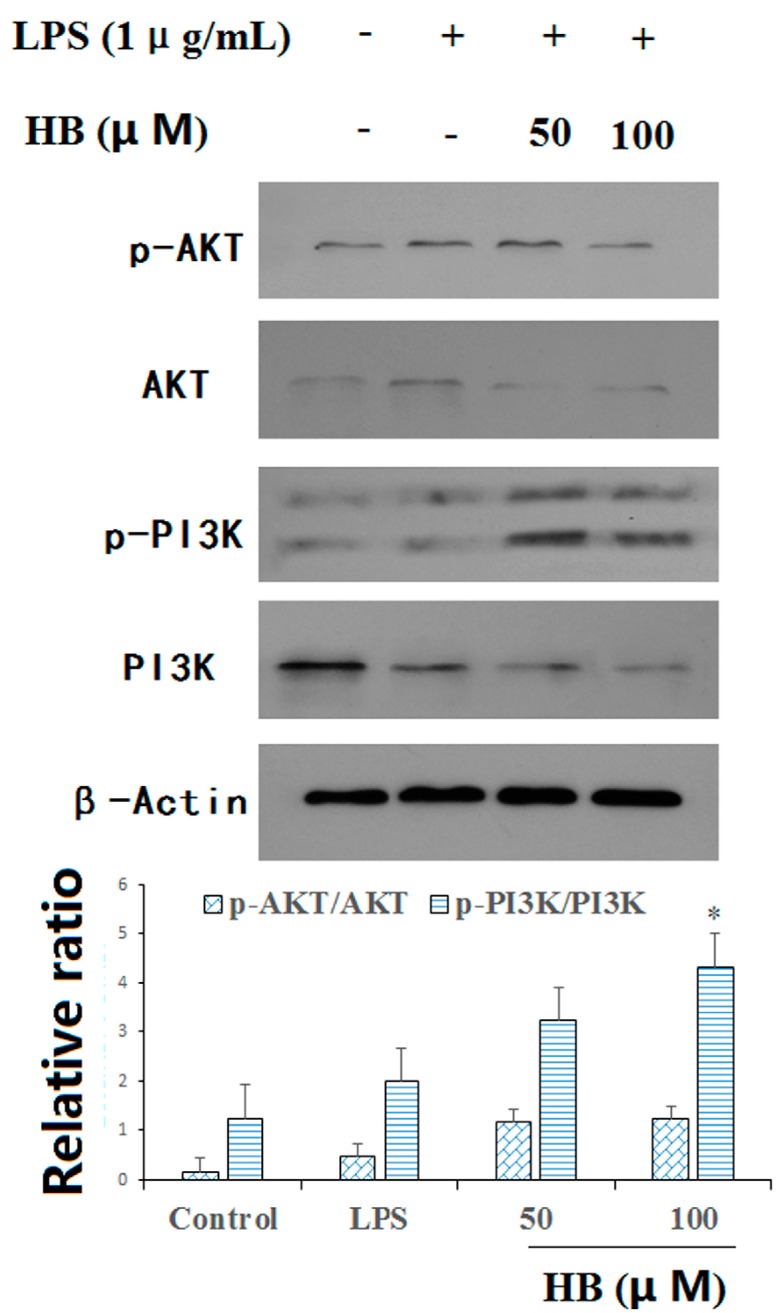
Effects of HB on LPS-induced PI3K and AKT phosphorylation. RAW 264.7 cells were pretreated with 50 and 100 μM HB for 1 h before treatment with 1 μg/mL LPS. After incubation for 30 min, the expression of levels of PI3K and AKT protein were measured by western blot analysis. The values are presented as means ± SD of three independent experiments. * *p* < 0.05, compared to the LPS-treated group.

We evaluated the effect of HB on LPS-induced phosphorylation of PI3K and AKT. Compared with the LPS group, HB could up-regulate the relative ratio of p-AKT/AKT. In addition, HB in a high concentration could significantly up-regulate the relative ratio of p-PI3K/PI3K (Figure 8).

## 3. Discussion

Macrophages play an important role in the activation and release of the pro-inflammatory mediators and cytokines, including NO, IL-1β, and IL-6, when treated by LPS. LPS is the thicker layer of lipopolysaccharide at the outer membrane of the cell wall of Gram-negative bacteria [10,11]. The process may be followed by oxidative stress [12] and apoptosis [13]. Therefore, we explored the treatment of HB on the inflammation, oxidative stress, and apoptosis in RAW 264.7 cells stimulated by LPS in the study.

HB, as one of the cyclic peptides of *Pseudostellaria heterophylla* (Miq.) Pax, has been received considerable attention for the treatment of tumor and inflammation. In China, *Pseudostellaria heterophylla* (Miq.) Pax, as a traditional Chinese medicine, has been often used in nourishing Yin [14], strengthening the spleen, replenishing Qi, moistening lungs, and producing fluids in Chinese medicine [15]. Previous studies showed that the extracts of the *Pseudostellaria heterophylla* (Miq.) Pax have the activity of anti-inflammatory [16]. However, there are few studies on the mechanism of its anti-inflammatory in vitro. The aim of the present research was to explore the anti-inflammatory activity of HB and clarify the underlying molecular mechanisms in RAW 264.7 cells.

NO is a major inflammatory mediator involved in various inflammatory diseases. In the research, the inhibitory activity of NO production was first estimated to test the anti-inflammatory activity. Next, pro-inflammatory cytokines, such as IL-1β and IL-6 are key mediators of inflammatory diseases. Pro-inflammatory cytokines can trigger the production of reactive oxygen species (ROS) [17]. Consequently, we explored whether HB suppressed the production of pro-inflammatory cytokines by ELISA and qRT-PCR. The results showed that HB suppressed the expression and mRNA levels of IL-6 and IL-1β in a dose-dependent manner in LPS-activated RAW 264.7 macrophages. Furthermore, HB suppressed the generation of ROS, as detected by flow cytometry. Our results also showed that HB exerts anti-inflammatory activity through the PI3K/AKT pathways.

The PI3K/AKT pathway has been shown to control a variety of cellular processes, including cell survival and proliferation. There are emerging therapeutic trials that target the PI3K/AKT pathway [18]. Furthermore, another research certified that activation of endogenous anti-oxidants to inhibit apoptosis is associated with Akt pathway activation [19]. It is well known that PI3K plays a core role in promoting the survival of a wide range of cell types, and that activation of Akt can promote cell proliferation and differentiation [20]. Emerging evidence suggests that the PI3K/AKT signaling pathway is a classical signal pathway in regulating cell survival [21,22]. The phosphoinositide 3-kinase/protein kinase B (PI3K/AKT) pathway is known as a central mediator in signal transduction pathways involved in cell growth, cell survival, and metabolism. Furthermore, PI3K/AKT activation has been shown to inhibit autophagy, which might contribute to the protection of mammalian cells against various damaging conditions.

## 4. Materials and Methods

### 4.1. Drugs and Chemicals

HB was obtained from Shanghai Yuanye Bio-Technology Co., Ltd. (≥98% purity; Shanghai, China). Dulbecco’s modified Eagle’s medium (DMEM) and fetal bovine serum (FBS) were purchased from Corning (Corning, NY, USA). LPS (from *E. coli*, isotype 055:B5) was obtained from Sigma Chemical Co. (St. Louis, MO, USA). PBS, dimethyl sulfoxide (DMSO) and 3-(4,5-dimethylthiazol-2-yl)-2,5-dipheny-ltetrazolium bromide (MTT) were obtained from Solarbio (Beijing, China). Nitric oxide assay kit was purchased from Applygen Co. (Beijing, China). IL-1β and IL-6 ELISA kits were obtained from BOSTER (Wuhan, China). Total RNA Extraction kit and One-step RT-PCR kit was obtained from Invitrogen (Carlsbad, CA, USA). The antibodies to AKT and PI3K, and the phosphorylated forms of AKT and PI3K were obtained from Cell Signaling Technology (Danvers, MA, USA). Polyvinylidene fluoride (PVDF) membrane and ECL western blotting detection reagent were obtained from Pierce Manufacturing (Appleton, WI, USA).

### 4.2. Cell Culture

RAW 264.7 cells (Beijing Union Medical University, Beijing, China) were maintained in high glucose DMEM medium supplemented with 10% heat-inactivated FBS and 1% penicillin (10,000 U/mL)-streptomycin (10,000 μg/mL) in a humidified atmosphere containing 5% CO_2_ at 37 °C.

### 4.3. Cell Viability Assay

The effect of HB on RAW 264.7 cells was assessed by MTT assay. Briefly, cells were plated into 96-well plates with a density of 3.0 × 10^4^ cells/well. After 24 h, the cells were treated with 0, 25, 50 and 100 μM HB for 24 h. DMSO (0.1%) was used as the untreated control. Then, 100 μL MTT working solution (0.5 mg/mL) was added. After incubation for 2–4 h at 37 °C, culture supernatant was removed from all the wells, and the purple formazan crystals were dissolved in 100 μL DMSO. Finally, a microplate reader (Multiskan GO, Thermo, Waltham, MA, USA) was used to measure the absorbance of the formazan solution at 570 nm.

### 4.4. NO Production Assay

Nitric oxide assay kit was used to detect the NO production in the culture medium based on the Griess reaction. RAW 264.7 cells (1 × 10^6^/well) were plated into 96-well plates and pretreated with 25–100 μM of HB for 1 h before treatment with 1 μg/mL LPS. After incubation for 18 h, the culture supernatant was mixed with an equal volume of Griess reagent. The absorbance of the mixture was read at 540 nm using a microplate reader.

### 4.5. ELISA Assays

RAW 264.7 cells were plated into 96-well plates with a density of 1.0 × 10^4^ cells/well and pretreated with 25–100 μM of HB for 1 h before treatment with 1 μg/mL LPS. After incubation for 18 h, the levels of IL-6 and IL-1β in the culture supernatant were measured using the corresponding ELISA kits following the manufacturer’s instructions. 

### 4.6. Measurement of Intracellular ROS

Because ROS is critical for LPS-induced inflammation by activation of Akt or NF-kB signaling [23], we carried out studies to measure the effects of HB on intracellular ROS accumulation. Generation of intracellular ROS was determined using the DCFH-DA fluorescent dye [24,25]. DCFH-DA probe, a non-polar compound which lightly diffuses into cells, is hydrolyzed by intracellular esterase to generate DCFH which is captured into the cells. Then, DCFH can be oxidized to form the highly fluorescent compound 2,7-dichlorofluorescein (DCF) which is measured by flow cytometry. In this assay, cells were seeded in 6-well plates at a density of 1 × 10^6^ cells/well exposed to HB (100 μM) for 1 h before treatment with 1 μg/mL LPS. After incubation for 18 h, the cells were incubated with 10 μM DCFH-DA for 30 min at 37 °C in the dark. Subsequently, the cells were harvested, washed twice with PBS, and re-suspended for analysis. The fluorescence was detected using flow cytometry.

### 4.7. Apoptosis Analysis

Apoptosis was detected using an Annexin V-FITC Detection Kit and determined by flow cytometry [26]. In brief, cells were plated in 6-well plate (1.0 × 10^6^ cells/well) and incubated with HB at doses of 100 μM for 1 h 37 °C before treatment with 1 μg/mL LPS. After incubation for 18 h, the cells were collected and washed with PBS. The cells were re-suspended in 295 μL binding buffer and incubated with 5 µL Annexin V-FITC and 10 µL PI at room temperature in the dark. Then, the cells were washed and re-suspended with PBS. All of the samples were instantly analyzed by flow cytometry (BD FACS Canto II, Franklin Lakes, NJ, USA).

### 4.8. qRT-PCR Analysis

The methods for total RNA extraction and quantitative real-time polymerase chain reaction (qRT-PCR) have been previously described [27]. In short, RAW 264.7 cells were plated into 6-well plates with a density of 1.0 × 10^6^ cells/well and pretreated with 50–100 μM of HB for 1 h before treatment with 1 μg/mL LPS. After treatment for 18 h, the cells were harvested and the total RNA was isolated by using Trigol reagent (Invitrogen, CA, USA). The β-actin acted as a control for total messenger RNA (mRNA) amount. The following PCR primer sequences (forward and reverse, respectively) were used: 5′-AGGTCGGTGTGAACGGATTTG-3′and 5′-GGGGTCGTTGATGGCAACA-3′ for GAPDH; 5′-CTGCAAGAGACTTCCATCCAG-3′ and 5′-AGTGGTATAGACAGGTCTGTTGG-3′ for IL-6; 5′-GAAATGCCACCTTTTGACAGTG-3′ and 5′-TGGATGCTCTCATCAGGACAG-3′ for IL-1β. Values were analyzed by normalizing with GAPDH mRNA expression. The mRNA expression was calculated using the 2^−ΔΔCt^ method. 

### 4.9. Western Blot Analysis

RAW 264.7 cells were seeded in 6-well plates and incubated with various concentrations of HB. Then the cells were collected and lysed with ice-cold RIPA buffer for 30 min. Subsequently, the lysates were centrifuged for 10 min at 12,000 rpm. A BCA protein assay kit was used to determine total protein concentration of the supernatant. In a parallel experiment, the mitochondrial and cytosolic fractions were separated by the ProteoExtract^®^ Cytosol/Mitochondria Fractionation Kit (Millipore, Darmstadt, Germany) in accordance with the manufacturer’s instructions. For sodium dodecyl sulfate-polyacrylamide gels (SDS-PAGE), an equal amount of target protein was loaded per lane and then transferred to a polyvinylidene fluoride (PVDF) membrane. The membranes were blocked with 5% skim milk in TBST (25 mM Tris, 150 mM NaCl, 0.1% Tween 20, pH 7.4) buffer for 1 h, and then incubated with primary antibodies overnight at 4 °C [28]. After being washed four times with TBST, the membranes were further incubated in corresponding secondary antibodies at room temperature for 1 h. The target proteins were visualized with an ECL western blotting detection reagent (Pierce, Appleton, WI, USA). Semi-quantitation of the scanned films was performed using Quantity One (Bio-Rad, Hercules, CA, USA). All the experimental results were repeated at least thrice.

### 4.10. Statistical Analysis

Each experimental result was repeated in triplicate and data were expressed as mean ± SD. Data were processed using 17.0 SPSS software (SPSS Inc., Chicago, IL, USA). Statistical significance was analyzed using One-Way ANOVA analysis and LSD test. A difference was considered significant when * *p* < 0.05, ** *p* < 0.01, and *** *p* < 0.001.

## 5. Conclusions

In the research, HB shows significant effects on the inhibition of pro-inflammatory mediators including NO and cytokine production, such as IL-1β and IL-6, in LPS-stimulated RAW 264.7 macrophages. In addition, HB suppressed the production of ROS and the apoptosis induced by LPS in RAW 264.7 macrophages. HB decreased the level of IL-1β and IL-6 expression by qRT-PCR analysis and up-regulated the relative ratio of p-AKT/AKT and p-PI3K/PI3K as indicated by western blot analysis. In summary, the results of study showed that HB were in line with the suppression of oxidative stress-triggered damage and inflammatory cytokines by the PI3K/Akt signaling pathway. Of course, the therapeutic effect of HB in vivo and the relationships of its inhibitory effects on other signaling pathways are needed for further studies.
